# Remote Glucose Monitoring Engagement and Glycemic Control in Type 2 Diabetes and Prediabetes: Retrospective Cohort Study

**DOI:** 10.2196/95679

**Published:** 2026-09-30

**Authors:** Yong Huang, Nitish Nagesh, Ajan Subramanian, Li Wang, Zhiyu Liu, Yichen Sun, Weiyi Hou, Iman Azimi, Amir M Rahmani, Randall S Stafford

**Affiliations:** 1 Department of Computer Science, Donald Bren School of Information and Computer Sciences, University of California, Irvine, 3211 Donald Bren Hall, Irvine, CA, 92617, United States, 1 8588881526; 2 Unika Health, Sunnyvale, CA, United States; 3 Nhu Department of Electrical Engineering and Computer Science, The Henry Samueli School of Engineering, University of California, Irvine, Irvine, CA, United States; 4 Sue & Bill Gross School of Nursing, University of California, Irvine, Irvine, CA, United States; 5 Stanford University, Palo Alto, CA, United States

**Keywords:** patient-centered care, type 2 diabetes mellitus, prediabetic state, digital health, remote patient monitoring, glucose monitoring, glycemic control, causal inference, marginal structural models

## Abstract

**Background:**

Suboptimal glycemic control remains a significant public health challenge among adults with type 2 diabetes and prediabetes, with 47.4% of US adults with diagnosed diabetes having hemoglobin A_1c_ (HbA_1c_) ≥7%. Remote glucose monitoring programs have shown promise for supporting self-management, but real-world evidence on the causal impact of varying patient engagement levels on glycemic outcomes remains limited.

**Objective:**

This study aimed to estimate the causal dose-response relationship between patient engagement, operationalized as weekly glucose monitoring frequency, and glycemic control measured by HbA_1c_ among adults enrolled in a comprehensive primary care–integrated remote monitoring program.

**Methods:**

We conducted a retrospective cohort study of 1436 adults with type 2 diabetes or prediabetes enrolled in the Unika Health program between 2019 and 2024. The program integrated Bluetooth-connected glucose meters, a mobile app, structured lifestyle coaching, and primary care coordination across 74 physician practices. Engagement was defined as mean weekly glucose monitoring frequency during the first 6 months. The causal effect of monitoring frequency on 6-month HbA_1c_ was estimated using marginal structural models (MSMs) with inverse probability weighting to address time-varying confounding. Covariates included age, sex, BMI, baseline HbA_1c_, comorbidities, medication status, and physical activity level.

**Results:**

The cohort was predominantly older (95.4%, 1370/1436 aged ≥46 years), with 82.6% (1186/1436) having hypertension and 45.9% (659/1436) classified as obese. Overall, HbA_1c_ decreased by a mean of 0.54 (SD 1.47; 95% CI 0.47‐0.62) percentage points (*P*<.001) over 6 months. Cluster analysis identified 3 engagement tiers: low (n=835; mean 2.55, SD 1.35 measurements/week), medium (n=493; mean 6.19, SD 1.46 measurements/week), and high (n=108; mean 12.59, SD 3.03 measurements/week). A monotonic dose-response was observed, with mean HbA_1c_ reductions of 0.38 (SD 1.40; 95% CI 0.29‐0.48), 0.71 (SD 1.50; 95% CI 0.58‐0.85), and 1.01 (SD 1.67; 95% CI 0.69‐1.32) percentage points for the low, medium, and high tiers, respectively (all *P*<.001 by 2-tailed paired *t* test). In weighted MSMs, each additional weekly measurement was associated with a 0.05 (95% CI 0.03‐0.07) percentage point greater HbA_1c_ reduction (*P*<.001). Among patients with high baseline HbA_1c_ (≥9%), the high-engagement group achieved a mean reduction of 3.12 (SD 1.97; 95% CI 2.29‐3.94) percentage points. Findings were consistent in sensitivity analyses at 3 months (β=−0.04; *P*<.001) and 12 months (β=−0.03; *P*=.003) and across alternative weighting specifications.

**Conclusions:**

Higher engagement with a digitally enabled, primary care–integrated remote glucose monitoring program was causally associated with significantly greater HbA_1c_ reductions in adults with type 2 diabetes and prediabetes. These findings support scalable remote patient monitoring strategies that actively foster sustained patient engagement as an effective approach to improving glycemic control and reducing the burden of diabetes-related complications at a population level.

## Introduction

Diabetes mellitus represents a growing global health challenge, affecting approximately 537 million adults as of 2021, with projections indicating an increase to 783 million by 2045 [1]. In the United States, 38.4 million Americans (11.6% of the population) have diabetes, with approximately 90% to 95% having type 2 diabetes, while an additional 97.6 million adults (38% of the population) have prediabetes. The condition imposes substantial economic and clinical burdens, with annual global expenditures estimated at US $966 billion and significant impacts on quality of life through complications including retinopathy, nephropathy, neuropathy, and cardiovascular disease [2]. Recent technological advances have transformed diabetes self-monitoring through digital glucose meters connected to mobile health apps, enabling real-time tracking and seamless data sharing with health care providers [3]. Large-scale studies have shown the effectiveness of self-monitoring of blood glucose (SMBG) in improving metabolic control and reducing hemoglobin A_1c_ (HbA_1c_) levels across diverse populations with varying preexisting conditions [4,5]. Contemporary remote patient monitoring (RPM) programs extend beyond simple data collection to include comprehensive care coordination, personalized coaching, and behavioral support. Modern RPM implementations integrate monitoring technology with multidisciplinary care teams including registered dietitians, certified diabetes educators, care coordinators, and health coaches who provide support between clinical visits, recognizing that sustained behavior change requires continuous engagement and adaptive coaching strategies. A growing body of evidence supports the effectiveness of RPM and related telehealth interventions for improving diabetes management. Systematic reviews show that RPM interventions generally produce modest but significant improvements in glycemic control, with HbA_1c_ reductions typically ranging from −0.3% to −0.5% [6-10].

However, critical gaps remain in RPM research, especially concerning patient engagement patterns and their causal relationship to clinical outcomes. While technology facilitates monitoring, consistent patient engagement, including regular measurements, medication use, app interaction, and communication, significantly influences outcomes [11,12]. These varied dimensions of engagement are likely interconnected and collectively contribute to the overall impact of an RPM program. Current literature suggests positive associations between engagement and glycemic control [13,14], but clear causal evidence is lacking, especially for dose-response relationships between varying levels of active participation and glycemic outcomes. Quantifying engagement effects from observational data requires addressing unique analytical challenges that traditional regression approaches cannot adequately handle. There is a need to quantify engagement effects and evaluate the causal relationship of RPM on glycemic outcomes to enable targeted patient treatment interventions. The aim of this study was to quantify the causal relationship between patient engagement, operationalized as remote glucose monitoring frequency, and glycemic control measured by HbA_1c_ among adults with type 2 diabetes and prediabetes enrolled in a comprehensive, primary care–integrated remote monitoring program.

## Methods

### Study Design, Setting, and Intervention Program

We conducted a retrospective cohort study analyzing data from patients with type 2 diabetes enrolled in the Unika Health program between 2019 and 2024. Each patient was added as soon as they enrolled in the program, and their blood glucose levels were monitored using the Gluco+ Wireless Smart Gluco-Monitoring System and their readings were subsequently accessed on a mobile app. Unika Health is a comprehensive digital health care delivery model that combines RPM, chronic-care management, and structured lifestyle coaching to extend care between physician encounters. The program was implemented in collaboration with 74 physician practices and organizations.

Within this model, an interdisciplinary team—including primary care physicians, registered dietitians, certified diabetes care and education specialists, and care coordinators—conducts initial enrollment, device onboarding, comprehensive baseline assessment, and collaborative goal setting. Physiologic and behavioral data are transmitted continuously; algorithm-defined thresholds prompt targeted outreach, supplemented by regular one-on-one dietitian counseling, physician follow-ups, and laboratory assessments every 3 to 6 months.

To comply with HIPAA (Health Insurance Portability and Accountability Act) deidentification standards, multiple safeguards were implemented to protect participant privacy. Member identifiers were encrypted using asymmetric key encryption, with a hashed version retained to maintain analytical consistency without revealing identity. All personally identifiable information, including names and direct identifiers, was removed. Temporal variables were uniformly shifted to mask exact dates while preserving seasonal and longitudinal patterns relevant for analysis. Numeric ages were converted into categorical groups, and race information was consolidated into broader categories to minimize reidentification risk. Together, these measures ensured secure, ethical, and reproducible handling of sensitive health data.

### Participants

To be included in the analysis, patients were required to meet the following criteria:

- A diagnosis of type 2 diabetes mellitus (n=13,155)
- A baseline HbA_1c_ measurement within the baseline window (between 6 months before enrollment and 1 month after enrollment, n=8853)
- An end point HbA_1c_ measurement at 6 months (+1 to –1 month) after enrollment (n=1801)
- At least one before-meal glucose measurement during the first 6 months of the study period (n=1436)

The requirement of at least one before-meal glucose measurement served as a minimum inclusion threshold to confirm device activation and program participation; it was not the engagement exposure itself. Engagement was operationalized separately as each patient’s mean weekly measurement frequency across the 6-month observation period. All readings were captured automatically from Bluetooth-connected glucose meters and were device time-stamped at the point of measurement rather than manually entered, and before-meal status was recorded as a structured field transmitted with each reading.

When multiple HbA_1c_ results were available within the baseline or end point windows, we selected the one closest to the enrollment date for baseline measurements and the one closest to the 6-month mark for end point measurements. Prestudy HbA_1c_ measurements were not accessible, as data from prior to enrollment reside in external sources outside of the program’s data infrastructure. Therefore, the first HbA_1c_ measurement recorded during the study period was designated as the baseline.

Participants were recruited through physician referrals to the Unika Health program. Eligible patients had a diagnosis of type 2 diabetes mellitus and were prescribed SMBG as part of their management plan.

Upon enrollment, participants received Bluetooth-connected glucose monitoring devices and access to a mobile app for tracking their measurements. The program collected several types of data:

- Blood glucose measurements: time-stamped readings from patient self-monitoring activities
- Laboratory tests: HbA_1c_ measurements from routine clinical care visits
- Demographic information: age, sex, BMI, physical activity levels, and other patient characteristics
- Clinical data: comorbidities (hypertension and hyperlipidemia), treatment type, and lifestyle factors (smoking and drinking status)

The primary outcome was the HbA_1c_ measurement taken in the end point window. As shown in Figure 1, the study timeline includes a baseline window for the initial HbA_1c_ measurement, a 6-month observation period for glucose monitoring, and an end point window for the final HbA_1c_ assessment.

**Figure 1.**
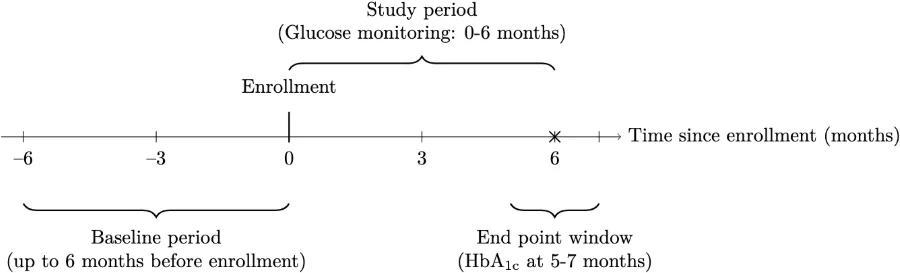
Study timeline showing the baseline window (6 months before to 1 month after enrollment), 6-month observation period for glucose monitoring, and end point window (5-7 months after enrollment) for hemoglobin A_1c_ (HbA_1c_) assessment.

### Remote Monitoring Frequency and Covariates

The primary focus of this study was remote monitoring frequency, quantified by the weekly frequency of self-administered glucose measurements. This continuous, time-varying metric served as our treatment variable within the causal inference framework, where higher frequencies indicated greater remote monitoring frequency levels. Monitoring frequency was calculated based solely on glucose measurements captured during the study period, as pre-enrollment monitoring data were unavailable. As this was a retrospective study, engagement was operationalized based on monitoring frequency captured via the connected blood glucose monitoring device; detailed analysis of app interaction features and cross-platform engagement was not conducted and was beyond the scope of this work.

Patient-specific baseline covariates included demographic characteristics (age at enrollment and sex), clinical parameters (BMI category and baseline HbA_1c_), comorbidity status, physical activity levels, and lifestyle factors (alcohol consumption and smoking status).

We identified several time-varying confounders based on their potential interactions with remote monitoring frequency: (1) weekly mean before-meal glucose readings, which reflect current glycemic control; (2) program duration in weeks, accounting for stage-specific intervention effects; and (3) weekly medication adherence status and medication type (oral, insulin, or others).

The primary outcome was defined as the HbA_1c_ value obtained at the follow-up lab visit after the 6-month study period, which provided a direct assessment of glycemic control achieved during the period of observed remote monitoring frequency behavior.

### Statistical Analysis

Our statistical analysis began with a descriptive characterization of the study population. First, we summarized baseline cohort characteristics, including demographic distributions and clinical features. To understand patterns of remote monitoring frequency, we then conducted cluster analysis on longitudinal measurement behavior data, which identified 3 distinct profiles: 3 tiers with increasing monitoring frequency from tier 1 to tier 3, where tier 1 represents low engagement, tier 2 indicates medium engagement, and tier 3 corresponds to high engagement.

To estimate the causal effect of remote monitoring frequency on glycemic control, we used a formal causal inference framework that accounts for the complex temporal relationships between variables. The causal graph was developed based on two sources: (1) established frameworks in the marginal structural model (MSM) literature for handling time-varying treatments and confounders [15], and (2) prior applications of MSM in diabetes care research, particularly studies examining medication adherence and glycemic outcomes [16]. While our study focused on remote monitoring frequency rather than medication adherence, we adopted a similar causal structure to account for time-varying confounding in diabetes self-management behaviors.

### MSMs

To address time-varying confounding, we implemented MSMs with inverse probability weighting (IPW). This approach allowed for a more accurate estimation of the causal effect of glucose monitoring on glycemic control, especially when factors that change over time—such as interim glucose readings or medication use—can both influence and be influenced by monitoring behavior [7].

MSMs include 2 main stages. First, we calculated statistical weights for each patient at each time point, reflecting how likely their observed monitoring behavior was, given their prior health status and characteristics. These weights helped balance the study population, making it more comparable to a randomized trial and reducing bias from time-varying confounders. To ensure stability, we limited the influence of extreme weights.

The weighting model included the following baseline covariates, selected for their clinical association with both monitoring behavior and glycemic outcome: age group, sex, BMI category, assigned treatment, baseline comorbidities (hypertension, hyperlipidemia, mixed hyperlipidemia, and diabetes complication categories), smoking status, drinking status, and physical activity level and frequency. The denominator model additionally included time-varying covariates—previous-week mean glucose, program week, and weekly medication status (oral, insulin, and other)—to address time-varying confounding. We assessed covariate balance before and after weighting using standardized mean differences (SMDs), with an SMD<0.1 considered balanced.

After weighting, the strongest measured confounder, baseline HbA_1c_, improved in balance (SMD reduced from 0.29 to 0.14), though several covariates retained SMDs above 0.1, indicating residual imbalance. Because balance was not fully achieved by weighting alone, baseline covariates were retained in the outcome model; this doubly robust specification yields a consistent estimate if either the weighting or the outcome model is correctly specified.

In the second stage, we used these weights in a regression model to estimate the effect of monitoring frequency on HbA_1c_, adjusting only for baseline characteristics such as age, sex, BMI, comorbidities, and treatment type. We specifically avoided adjusting for variables measured after the start of monitoring, as this could distort the estimated effect.

Because it is not practical to model every possible monitoring pattern, we summarized each patient’s monitoring behavior using their average weekly frequency, following conventions in MSM literature [15]. All analyses were conducted using Python (version 3.8; Python Software Foundation) and the statsmodels (version 0.13.2) package [17].

### Ethical Considerations

This study constituted a secondary analysis of preexisting data originally collected by the Unika Health program during routine clinical care and program operations. The data were fully deidentified and anonymized in accordance with HIPAA deidentification standards. The research team at the University of California, Irvine was granted explicit permission to access and use the deidentified dataset for research purposes under a data use agreement with Unika Health, ensuring the data were used solely for the research purposes intended. Informed consent was obtained from all participants as part of their original enrollment in the Unika Health program. Patient privacy and confidentiality were maintained throughout, and no identifying details were published in this manuscript.

## Results

### Participant Characteristics

A total of 1436 patients with type 2 diabetes or prediabetes comprised the study cohort (Table 1). The cohort had a nearly equal gender distribution (50.1%, 719/1436 female) and was predominantly older, with 95.4% (1370/1436) aged 46 years or older. Nearly half (45.9%, 659/1436) were classified as obese, with an additional 31.8% (456/1436) as overweight. Hypertension was present in 82.6% (1186/1436) of the patients, and hyperlipidemia in 47.7% (685/1436), with 25.9% (372/1436) having mixed hyperlipidemia.

**Table 1.** Baseline characteristics of study participants (N=1436).

Characteristic	Participants, n (%)
Sex	
Male	717 (49.9)
Female	719 (50.1)
Age (y)	
18-25	1 (0.1)
26-45	65 (4.5)
46-65	463 (32.2)
66-80	749 (52.2)
81-89	124 (8.6)
≥90	34 (2.4)
BMI category	
Underweight	23 (1.6)
Healthy weight	291 (20.3)
Overweight	456 (31.8)
Obesity	659 (45.9)
Comorbidities^a^	
Hypertension	1186 (82.6)
Hyperlipidemia	685 (47.7)
Mixed hyperlipidemia	372 (25.9)
Diabetes complications^a^	
No complications	715 (49.8)
Kidney complications	231 (16.1)
Eye complications	56 (3.9)
Neurological complications	193 (13.4)
Circulatory complications	31 (2.2)
Other specified complications	450 (31.3)
Unspecified complications	71 (4.9)
Smoking status	
Never	1275 (88.8)
Current	87 (6.1)
Former	74 (5.2)

a Categories are not mutually exclusive; 252 participants (17.5%) had more than one diabetes-complication category, and 12 (0.8%) had none recorded.

### Engagement Patterns and Glycemic Outcomes

The study cohort had a mean weekly glucose measurement frequency of 4.55 (SD 3.25) measurements (median 3.82, IQR 2.00-6.22). Cluster analysis identified three engagement profiles with distinct measurement patterns: the high-engagement group (7.5%, 108/1436) averaged 12.59 (SD 3.03) measurements/week, the medium-engagement group (34.3%, 493/1436) averaged 6.19 (SD 1.46), and the low-engagement group (58.1%, 835/1436) averaged 2.55 (SD 1.35). Figure 2 illustrates the longitudinal trends in home glucose readings across these engagement groups.

**Figure 2.**
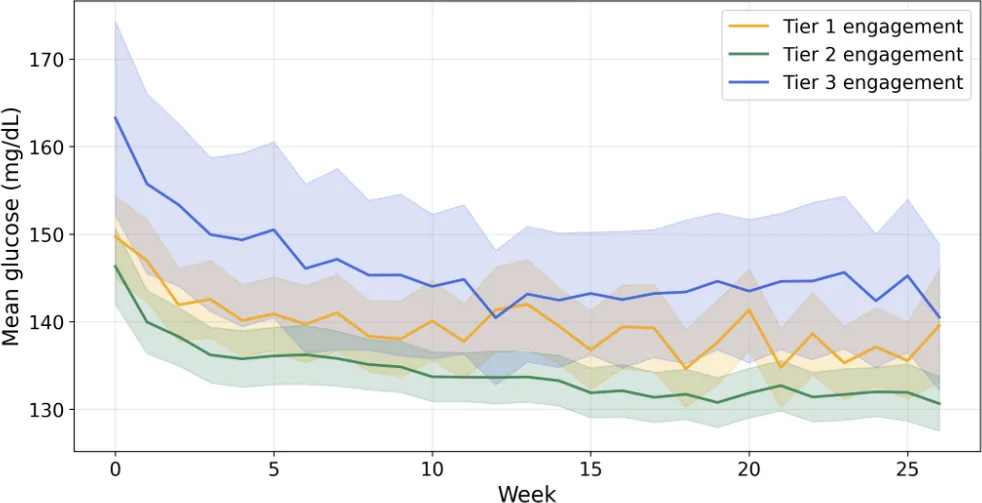
Mean weekly blood glucose (mg/dL) over the 6-month observation period by engagement tier. Tier 3 represents the high-engagement group. Measurement frequency over time is shown separately in Figure 3.

**Figure 3.**
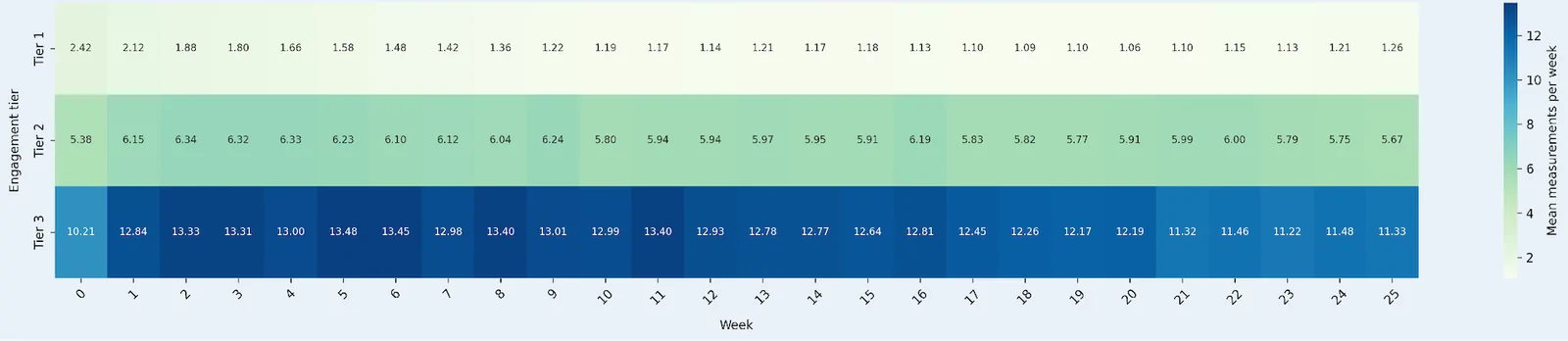
Mean weekly glucose measurement frequency by engagement tier across the observation period. Cell values are the mean measurements per patient per week within each tier. Monitoring frequency was highest in the opening weeks and declined modestly thereafter in all tiers.

Monitoring frequency was highest in the opening weeks of the program and declined modestly thereafter in all tiers (Figure 3). The high-engagement group began at approximately 10 to 13 measurements per week and stabilized near 11 to 13 measurements per week; the medium-engagement group remained near 6 measurements per week; and the low-engagement group declined from roughly 2.4 to about 1.1 measurements per week. As no pre-enrollment meter data were available, “baseline” frequency refers to the initial weeks of the observation period rather than a preprogram measurement.

Glycemic control improved across all engagement tiers, with a monotonic dose-response gradient in HbA_1c_ reduction (Table 2). The high-engagement group achieved the greatest mean reduction (1.01, SD 1.67 percentage points; 95% CI 0.69-1.32), followed by the medium-engagement (0.71, SD 1.50; 95% CI 0.58-0.85) and low-engagement (0.38, SD 1.40; 95% CI 0.29-0.48) groups. All three within-patient reductions were statistically significant (all *P*<.001, 2-tailed paired *t* test), with 95% CIs excluding zero. The high-engagement group also presented with the highest baseline HbA_1c_ (mean 8.04%, SD 1.76), consistent with greater room for improvement.

**Table 2.** Glycemic control outcomes by engagement tier.

Tier	Participants, n	Baseline HbA_1c_^a^ (%), mean (SD; 95% CI)	End point HbA_1c_ (%), mean (SD; 95% CI)	HbA_1c_ reduction^b^ (percentage points), mean (SD; 95% CI)	*P* value^c^
Tier 1 (low)	835	7.53 (1.72; 7.42-7.65)	7.15 (1.34; 7.06-7.24)	0.38 (1.40; 0.29-0.48)	<.001
Tier 2 (medium)	493	7.62 (1.72; 7.47-7.77)	6.91 (1.08; 6.81-7.00)	0.71 (1.50; 0.58-0.85)	<.001
Tier 3 (high)	108	8.04 (1.76; 7.71-8.38)	7.04 (1.23; 6.81-7.27)	1.01 (1.67; 0.69-1.32)	<.001
Overall	1436	7.60 (1.73; 7.51-7.69)	7.06 (1.25; 6.99-7.12)	0.54 (1.47; 0.47-0.62)	<.001

a HbA_1c_: hemoglobin A_1c_.

b Within-patient change (baseline–end point).

c 2-tailed paired *t* test of baseline vs end point HbA_1c_.

Among patients with high baseline HbA_1c_ (≥9%; n=248), engagement patterns showed even more pronounced effects on glycemic control (Table 3). The high-engagement group demonstrated the highest control rate, defined as achieving HbA_1c_<7% at the 6-month end point (36.4%, 8/22), and the greatest mean reduction in HbA_1c_ (3.12, SD 1.97 percentage points). In contrast, the low-engagement group showed the lowest control rate (21.8%, 31/142) and the smallest mean reduction (2.17, SD 1.98 percentage points).

**Table 3.** Outcomes for patients with high baseline HbA_1c_^a^ (≥9%; N=248).

Tier	Participants, n	Achieved control^b^, n (%)	Baseline HbA_1c_ (%), mean (SD)	End point HbA_1c_ (%), mean (SD)	HbA_1c_ reduction (percentage points), mean (SD; 95% CI)	*P* value^c^
Tier 1 (low)	142	31 (21.8)	10.61 (1.50)	8.44 (1.80)	2.17 (1.98; 1.84-2.50)	<.001
Tier 2 (medium)	84	27 (32.1)	10.71 (1.58)	7.86 (1.63)	2.85 (2.27; 2.36-3.33)	<.001
Tier 3 (high)	22	8 (36.4)	10.93 (1.56)	7.81 (1.55)	3.12 (1.97; 2.29-3.94)	<.001

a HbA_1c_: hemoglobin A_1c_.

b HbA_1c_<7% at the 6-month end point.

c 2-tailed paired *t* test of baseline vs end point HbA_1c_.

### Engagement Effects Estimated by MSM

The treatment model produced stabilized weights with a mean of 1.05 (SD 0.46) and a median of 1.03 (IQR 0.87-1.16; range 0.09-12.94), indicating a well-behaved distribution centered near 1.0. The treatment model achieved an R^²^ of 0.63.

### Sensitivity Analysis

To examine whether the effect of monitoring engagement on glycemic control is consistent across different end point time points, we additionally analyzed outcomes at 3 months and 12 months. Table 4 presents the glycemic outcomes by engagement tier at each time point.

**Table 4.** Glycemic outcomes by engagement group at different time points.

Time point and tier		Participants, n (%)	Baseline HbA_1c_^a^ (%), mean (SD; 95% CI)	End point HbA_1c_ (%), mean (SD; 95% CI)	HbA_1c_ reduction^b^ (percentage points), mean (SD; 95% CI)
3 months (n=1875)					
	Low	1094 (58.3)	7.64 (1.75; 7.54-7.75)	7.23 (1.33; 7.15-7.31)	0.42 (1.30; 0.34-0.50)
	Medium	648 (34.6)	7.80 (1.74; 7.66-7.93)	7.01 (1.04; 6.93-7.09)	0.79 (1.45; 0.68-0.90)
	High	133 (7.1)	8.14 (1.87; 7.83-8.46)	7.04 (1.01; 6.86-7.21)	1.11 (1.70; 0.82-1.40)
	Overall	1875 (100)	7.73 (1.76; 7.65-7.81)	7.14 (1.22; 7.08-7.19)	0.60 (1.40; 0.53-0.66)
12 months (n=792)					
	Low	439 (55.4)	7.66 (1.91; 7.48-7.84)	7.13 (1.38; 7.00-7.26)	0.53 (1.75; 0.37-0.70)
	Medium	302 (38.1)	7.65 (1.64; 7.47-7.84)	6.91 (0.99; 6.80-7.02)	0.75 (1.46; 0.58-0.91)
	High	51 (6.4)	7.82 (1.56; 7.39-8.25)	7.01 (1.07; 6.72-7.30)	0.81 (1.50; 0.40-1.22)
	Overall	792 (100)	7.67 (1.79; 7.54-7.79)	7.04 (1.23; 6.95-7.12)	0.63 (1.63; 0.52-0.75)

a HbA_1c_: hemoglobin A_1c_.

b All within-tier reductions *P*<.001 (2-tailed paired *t* test).

The dose-response relationship between monitoring frequency and glycemic control remained consistent across time points. At 3 months, each additional weekly measurement was associated with a 0.04 (95% CI 0.03-0.05) percentage point reduction in HbA_1c_ (β=–0.04; *P*<.001), and at 12 months with a 0.03 (95% CI 0.01-0.06) percentage point reduction (β=–0.03; *P*=.003).

## Discussion

### Main Findings

Our analysis of 1436 patients with type 2 diabetes and prediabetes enrolled in the Unika Health RPM program demonstrated that higher glucose monitoring adherence is causally associated with substantial improvements in glycemic control, as measured by HbA_1c_ reduction. Consistent with our primary objective of quantifying the impact of varying engagement levels on glycemic outcomes in a real-world setting, we identified a clear dose-dependent relationship: each additional weekly glucose measurement was associated with a 0.05 percentage point decrease in HbA_1c_ (*P*<.001). Patients in the highest engagement group (mean 12.59 measurements/week) experienced a mean HbA_1c_ reduction of 1.01 percentage points, compared with 0.38 in the lowest engagement group (mean 2.55 measurements/week)—a difference of 0.63 percentage points—with the intermediate group showing a progressively greater improvement (Table 2). These findings were robust after adjustment for baseline confounders using MSM with IPW.

### Interpretation, Implications, and Comparison to Existing Literature

The observed dose-response relationship underscores the importance of sustained patient engagement in digital health interventions for diabetes management. Prior studies evaluating RPM programs have reported HbA_1c_ reductions of 0.28 to 0.54 percentage points compared with usual care [7-10]; however, these studies have largely treated engagement as a binary variable—enrolled versus not enrolled—rather than characterizing the gradient of active participation. Our work provides the first rigorous quantification of how incremental increases in remote monitoring frequency translate to measurable glycemic improvements in a large real-world cohort, advancing beyond general program efficacy to isolate the specific contribution of engagement intensity.

Notably, even patients in the low-engagement group experienced a meaningful 0.38 percentage point reduction in HbA_1c_ at the 6-month end point. This finding suggests that enrollment in a comprehensive, primary care–integrated RPM program may confer benefits beyond the direct effects of glucose monitoring frequency alone. The increased self-awareness, behavioral modifications, and access to personalized health coaching associated with program participation likely contribute to positive outcomes independent of monitoring frequency, consistent with theoretical frameworks of self-management support in chronic disease [18,19].

The engagement patterns observed in our study—ranging from 2.55 to 12.59 measurements per week—reflect the wide variability in SMBG adherence documented in national surveys, where many patients monitor less frequently than clinically recommended [20]. Our results suggest that even within this variability, a graded and meaningful glycemic benefit exists, reinforcing the clinical value of supporting any level of engagement while prioritizing strategies to increase monitoring frequency.

Our adjusted models further confirmed that baseline HbA_1c_, age, treatment modality, and the presence of complications were independently associated with glycemic control during program participation (Table 3) [21,22], consistent with established diabetes risk profiles. Additionally, longer program duration was independently associated with a modest but statistically significant decline in HbA_1c_, suggesting that sustained participation yields cumulative benefits beyond initial engagement levels.

Collectively, these findings indicate that the intervention’s impact derives from both the supportive, primary care–integrated RPM structure and the degree of active patient engagement. The intervention’s grounding in established clinical workflows and physician oversight aligns with current guidelines for diabetes management [23], and its remote delivery model extends access to ongoing, proactive guidance in real-world settings.

### Limitations

Several limitations should be acknowledged. First, despite adjustment for a broad set of confounders and the use of MSMs with IPW, unmeasured confounding cannot be completely ruled out. Factors such as diabetes education, health literacy, psychological factors, social support, and health care access may influence both monitoring frequency and glycemic outcomes. Additionally, we lacked complete patient histories and comorbidity data due to information residing in disparate electronic medical records to which we did not have direct access.

Second, our measure of engagement captured monitoring frequency but not the timing, quality, or clinical response to individual readings. We could not assess whether patients monitored at optimal time points or acted upon their glucose values. While digital glucose meters were used, the possibility of measurement error or data entry issues could not be entirely excluded.

Third, while medication status across oral agents, insulin, and other therapies was incorporated as a time-varying covariate in the weighting model, we did not have sufficiently granular data on therapy intensification or regimen switching within each engagement tier. Residual confounding by unmeasured treatment changes therefore cannot be excluded.

Fourth, measured engagement with the monitoring program may also correlate with other unmeasured positive health behaviors—such as increased physical activity or dietary improvement—that could independently contribute to the observed outcomes, precluding full attribution of glycemic benefit to monitoring frequency alone.

Fifth, our study population consisted of patients enrolled in a digital health program, who tend to be younger, more educated, and more technologically comfortable than the broader diabetes population. Generalizability to RPM programs with substantially different structures, intensities, or patient populations may therefore be limited.

### Conclusions

Our findings demonstrate that higher engagement with a digitally enabled, primary care–integrated RPM program is causally associated with substantially greater glycemic improvement among patients with type 2 diabetes and prediabetes. Beyond confirming program efficacy, this work highlights that the magnitude of benefit is meaningfully modifiable through engagement intensity—a finding with direct clinical and policy relevance. Given that suboptimal glycemic control remains a significant public health challenge, and that diabetes is a potent modifiable risk factor for cardiovascular disease, kidney disease, neuropathy, and retinopathy, scalable strategies that both expand access to integrated RPM programs and actively foster sustained patient engagement have the potential to meaningfully reduce the burden of diabetes-related complications at a population level.

Future work should extend beyond intermittent glucose monitoring to continuous glucose monitoring (CGM), which enables real-time, granular tracking without the limitations of single-point measurements. Rigorous clinical trials validating CGM integration within coordinated care delivery models—including hospital and clinical settings—are warranted. Additional research directions include leveraging AI-enabled devices for targeted behavioral interventions and analyzing text- and video-based coaching interactions to enable increasingly personalized care. Such advances, embedded within the supported self-management framework demonstrated here, may further amplify the benefits of digitally enabled diabetes care.

## Abbreviations

CGM: continuous glucose monitoring
HbA_1c_: hemoglobin A_1c_
HIPAA: Health Insurance Portability and Accountability Act
IPW: inverse probability weighting
MSM: marginal structural model
RPM: remote patient monitoring
SMBG: self-monitoring of blood glucose
SMD: standardized mean difference
